# Oxygen and carbon isotope variations in *Chamelea gallina* shells: Environmental influences and vital effects

**DOI:** 10.1111/gbi.12526

**Published:** 2022-09-26

**Authors:** Arianna Mancuso, Ruth Yam, Fiorella Prada, Marco Stagioni, Stefano Goffredo, Aldo Shemesh

**Affiliations:** ^1^ Marine Science Group, Department of Biological, Geological and Environmental Sciences University of Bologna Bologna Italy; ^2^ Fano Marine Center, The Inter‐Institute Center for Research on Marine Biodiversity Resources and Biotechnologies Fano Italy; ^3^ Department of Earth and Planetary Sciences Weizmann Institute of Science Rehovot Israel; ^4^ Environmental Biophysics and Molecular Ecology Program, Department of Marine and Coastal Sciences, Rutgers The State University of New Jersey New Brunswick New Jersey USA; ^5^ Marine Biology and Fisheries Lab, Dept. of Biological, Geological and Environmental Sciences University of Bologna Bologna Italy

**Keywords:** Adriatic Sea, bivalve, latitudinal gradient, shell stable isotopes, vital effect

## Abstract

Stable isotopes in mollusc shells, together with variable growth rates and other geochemical properties, can register different environmental clues, including seawater temperature, salinity and primary productivity. However, the strict biological control over the construction of biominerals exerted by many calcifying organisms can constrain the use of these organisms for paleoenvironmental reconstructions. Biologically controlled calcification is responsible for the so called vital effects that cause a departure from isotopic equilibrium during shell formation, resulting in lower shell oxygen and carbon compared to the equilibrium value. We investigated shell oxygen and carbon isotopic composition of the bivalve *Chamelea gallina* in six sites along with a latitudinal gradient on the Adriatic Sea (NE Mediterranean Sea). Seawater δ^18^O and δ^13^C_DIC_ varied from North to South, reflecting variations in seawater temperature, salinity, and chlorophyll concentration among sites. Shell δ^18^O and δ^13^C differed among sites and exhibited a wide range of values along with the ~400 km latitudinal gradient, away from isotopic equilibrium for both isotopes. These results hampered the utilization of this bivalve as a proxy for environmental reconstructions, in spite of *C. gallina* showing promise as a warm temperature proxy. Rigorous calibration studies with a precise insight of environment and shell growth are crucial prior to considering this bivalve as a reliable paleoclimatic archive.

## 1 INTRODUCTION

Marine calcifying organisms can be considered valuable recorders of past environmental change in marine habitats (Bemis et al., 1998; Chauvaud et al., 2005; Jones et al., 2009; Rhoads & Lutz, 1980; Schöne & Gillikin, 2013; Vihtakari et al., 2017). In particular, mollusc shells are potential paleo‐environmental archives due to their seasonal deposition of carbonate material, retaining high resolution temporal records of the ambient physical and chemical conditions during growth that can be detected by shell oxygen and carbon isotope composition (Klein et al., 1996a; Purroy et al., 2018; Schöne et al., 2003).

In molluscs, calcification occurs within the extrapallial fluid (EPF), which is secreted by the mantle and is isolated from seawater (Wheeler, 1992). The composition of the EPF might be significantly altered with respect to seawater due to the influence of mantle metabolic activity or to the contribution of carbon from metabolic sources (Klein et al., 1996b; Tanaka et al., 1986). As a consequence of such processes, termed “vital effects” and classified as kinetic or metabolic isotope effects, biomineral compositions may depart from isotopic equilibrium. (McConnaughey, 1989a, 1989b). Thus, developmental or ontogenetic changes can obscure environmental signals, such as oxygen and carbon isotopic equilibrium fractionation, that occur in some species (Gillikin et al., 2007; McConnaughey, 1989a; Schöne, 2008). Furthermore, shell growth relies on various environmental factors, including temperature, salinity and food availability, that are responsible for varying biomineralization rates and shell growth cessation when beyond the environmental optimum of the organism (Leng & Lewis, 2016; Schöne, 2008). Therefore, a detailed understanding of the physiology and growth rates of the organism that produces the mineralized structures is crucial to obtaining a reliable reading of geochemical signals from mollusc shells.

Oxygen (δ^18^O derived from ^18^O/^16^O ratios) and carbon (δ^13^C derived from ^13^C/^12^C ratios) isotopic composition of marine mollusc carbonates are robust proxies for seawater temperature and dissolved inorganic carbon, respectively (Bemis & Geary, 1996; Elliot et al., 2003; Goodwin et al., 2001; Hickson et al., 1999; Krantz et al., 1987). Shell oxygen isotope composition (δ^18^O_shell_) is a function of temperature, salinity and the oxygen isotope composition of seawater (δ^18^O_sw_) at the time of precipitation (Craig, 1965; Epstein & Mayeda, 1953). The temperature dependence of δ^18^O fractionation in biogenic carbonates has been linked to species‐specific vital effects (Bemis et al., 1998; Böhm et al., 2000; Wefer & Berger, 1991). Shell carbon isotope composition (δ^13^C_shell_) is determined by the isotopic composition of the dissolved inorganic carbon in seawater (δ^13^C_DIC_) and by the proportion of metabolic carbon involved in the calcite/aragonite precipitation (Sadler et al., 2012). The amount of metabolic respiratory CO_2_ incorporated into the skeleton is species‐dependent, varying from less than 10% to over 35%, and it can be high enough to overshadow the δ^13^C_DIC_ signal (Gillikin et al., 2005, 2006; Klein et al., 1996b; Lorrain et al., 2004).

While the incorporation of respired CO_2_ within the body of an organism is linked to metabolic effects (McConnaughey et al., 1997), kinetic effects are specifically associated with processes such as shell crystal growth rate, hydration and hydroxylation of CO_2_ in solution (McConnaughey, 1989b) and the isotope fractionation between species of δ^13^C_DIC_, which are present in the calcifying fluids (Adkins et al., 2003; Spero et al., 1997; Tripati et al., 2010; Zeebe, 1999). Taking into account the amplitude of the biologically induced fractionation, in addition to environmental conditions, is crucial for a reliable interpretation of δ^18^O and δ^13^C signatures from molluscs specimens.

The present study aimed to investigate shell δ^18^O and δ^13^C in the clam *Chamelea gallina* along with a latitudinal gradient in the Adriatic Sea (~400 km). Shell isotopic profiles of *C. gallina* were also investigated to study ontogenetic variations in shell δ^18^O and δ^13^C along with the gradient. This study also monitored seawater δ^18^O and δ^13^C_DIC_ along with the Adriatic Sea latitudinal gradient, where the presence of Po river delta plays a crucial role in the biogeochemical processes of this basin. The Po is the largest Italian river in terms of both length (652 km long) and average discharge (1500 m^3^ s^−1^; Montanari, 2012), supplying over 50% of freshwater input to the Northern Adriatic basin (Degobbis et al., 1986) and about 20% of total river discharge in the Mediterranean Sea (Russo & Artegiani, 1996). Fluvial δ^13^C_DIC_ has usually lower isotopic composition than oceanic due to the presence of CO_2_ originated from the decomposition of terrestrial vegetation (McConnaughey & Gillikin, 2008). In the Po Plain‐Adriatic Sea system, a strong geomorphological change took place during the Holocene in response to the glacioeustatic sea‐level variations (Amorosi et al., 2019). In the past, the North Western Adriatic coastal area was characterized by estuary systems, bounded seaward by a series of sandbars that isolated coastal lagoons and limited riverine plumes into the Adriatic (Amorosi et al., 2019). This past more stable shoreface depositional setting due to reduced influence of riverine plumes, warmer temperature and higher aragonite saturation state than today seemed to reduce the thermodynamic work required for organisms to deposit calcium carbonate (Hall‐Spencer & Harvey, 2019), making the calcification less expensive in terms of metabolic cost (Cheli et al., 2021; Clarke, 1993). Nowadays, the western Adriatic basin is characterized by the northern area with shallow continental shelf, representing the result of several southward progradations driven by sea level cycles, by the central Adriatic Sea, a small remnant basin reaching 260 m water depth and confined to the north by the Po River delta formed during the last sea level lowstand and by the Southern Adriatic Sea, originated as a consequence of the interaction between mass transport processes and deep water circulation (Ridente et al., 2007, 2008). The Adriatic Sea is a dynamic environment influenced by terrestrial, atmospheric and oceanic processes that provide many challenges to understanding these systems (Canuel et al., 2012; Pérez et al., 2016). In addition to environmental drivers such as freshwater discharge and coastal upwelling, it can be impacted, as the other coastal zones, by increases in atmospheric *p*CO_2_ with the alteration of the distribution of reactive inorganic carbon species, thus reducing pH values and the saturation state for calcium carbonate minerals (Ω) (Feely et al., 2004; Gazeau et al., 2007; Harris et al., 2013; Pérez et al., 2016) and anthropogenic change of river basins that further influence the natural export of water, nutrients, and carbon to estuarine and coastal marine ecosystems (Pérez et al., 2016; Regnier et al., 2013). The carbonate chemistry of riverine‐influenced near shore environments is, therefore, affected by lower salinity and resultant decreased alkalinity, eutrophication and resultant production/respiration cycles (Pérez et al., 2016; Salisbury et al., 2008).

This study covers several sites along with the latitudinal gradient in the Adriatic Sea and will contribute to the understanding whether vital/ontogenic process or environmental conditions govern the shell isotopic signature in the bivalve *C. gallina*, giving insight into the use of bivalve archives as providers of environmental information.

## 2 MATERIALS AND METHODS

### 2.1 Sample collection and treatment

**FIGURE 1:**
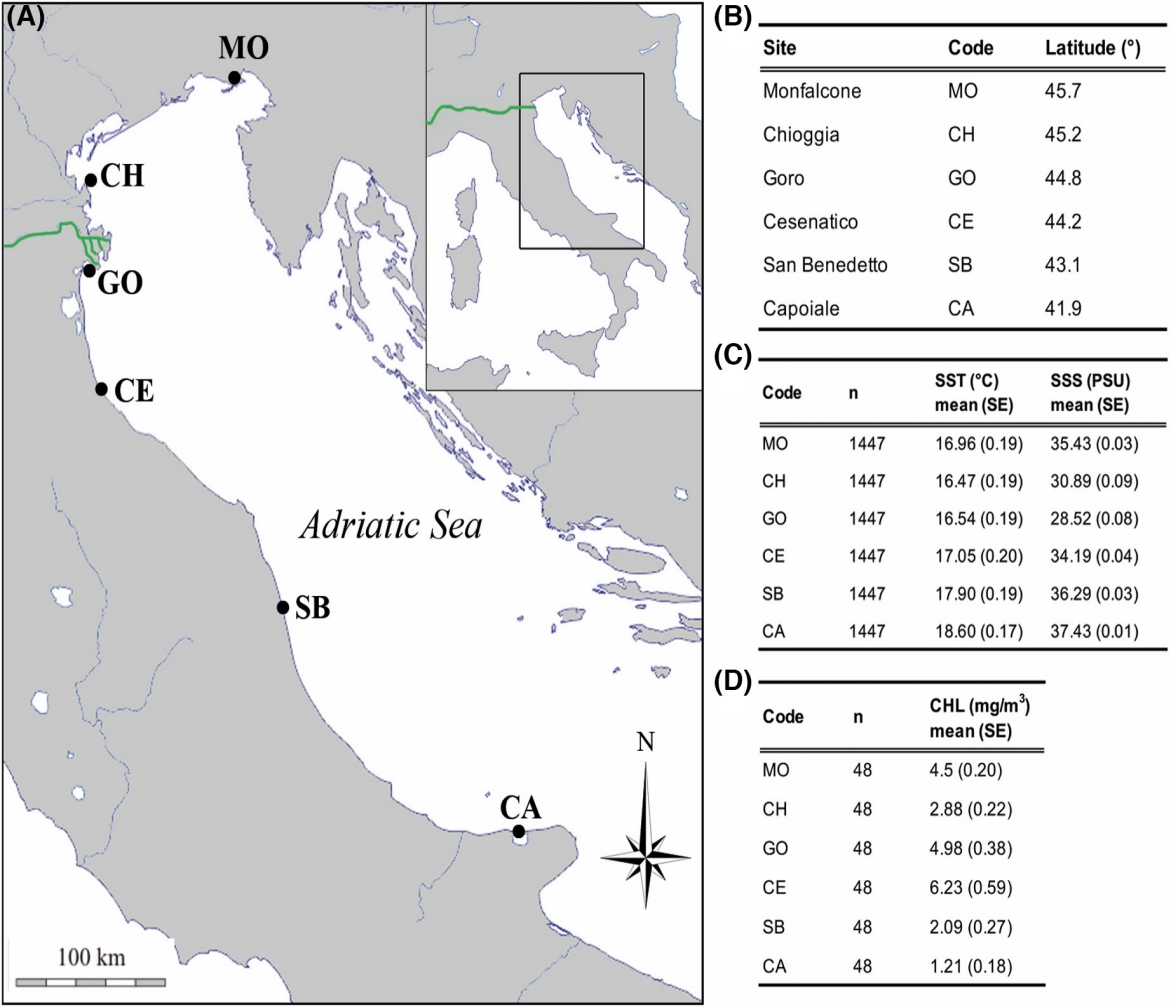
Map of the Adriatic Sea and environmental parameters. (A) Adriatic coastline with sampling sites of *C. gallina* clams. Po river labelled with green lines. Abbreviations and coordinates of the sites in decreasing order of latitude: MO, Monfalcone 45°42′N, 13°14′E; CH, Chioggia 45°12′N, 12°19′E; GO, Goro 44°47′N, 12°25′ E; CE, Cesenatico 44°11′N, 12°26′ E; SB, San Benedetto 43°5′N, 13°51′ E; CA, Capoiale 41°55′N, 15°39′ E. The map was downloaded from d‐maps.com site (http://www.d‐maps.com) and modified with Adobe Photoshop CS4. (B–D) Latitude, annual average values for sea surface temperature (SST), sea surface salinity (SSS) and chlorophyll concentration (CHL) from 2011 to 2015. n = number of collected data, daily data for SST and SSS and monthly data for CHL; SE = standard error.

**FIGURE 2:**
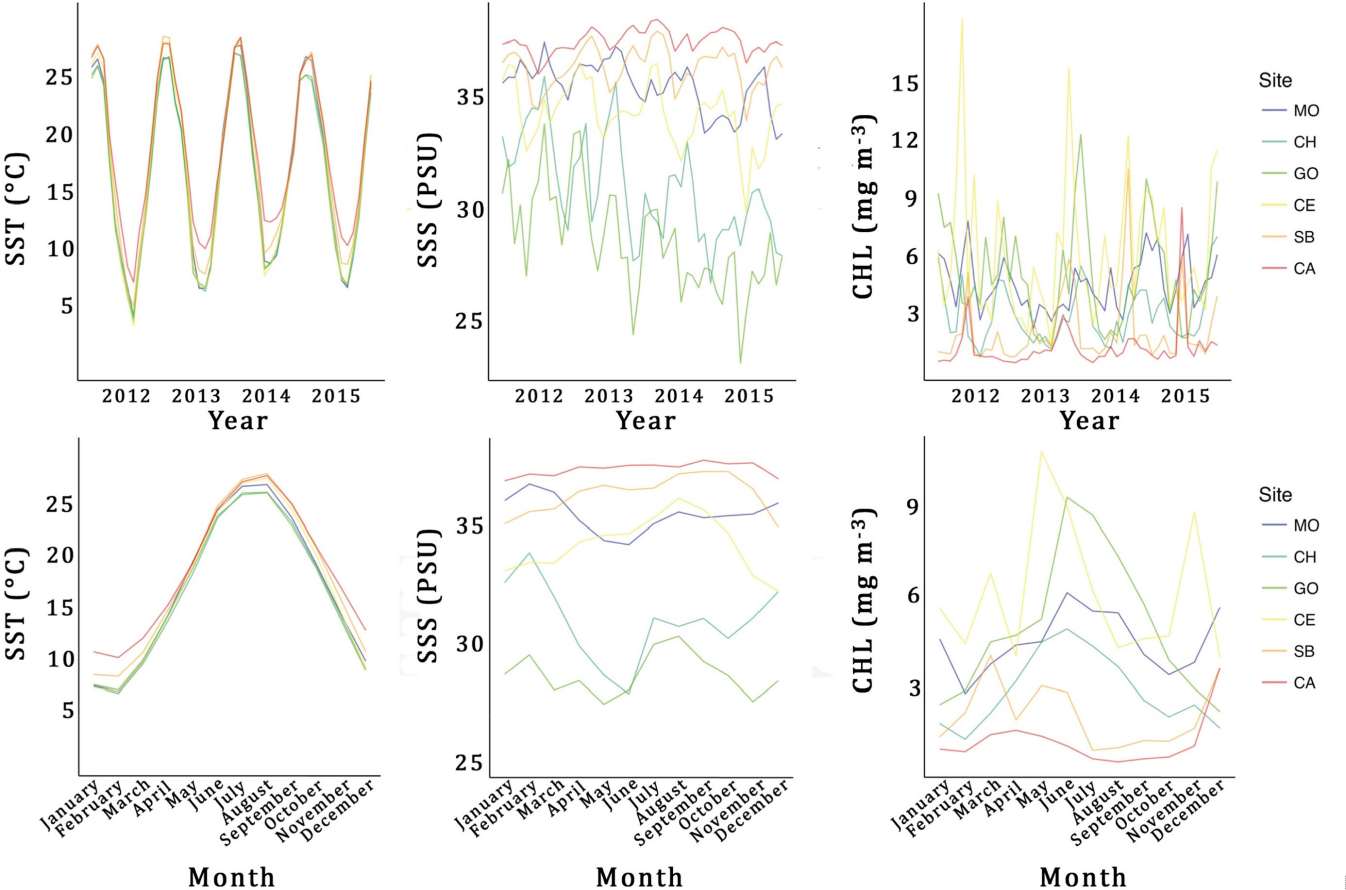
Inter‐annual and intra‐annual variations of SST, SSS and CHL among sites. Inter‐annual data from July 2011 to June 2015. Intra‐annual data are the mean monthly values of 4 years. Abbreviations in decreasing order of latitude: MO, Monfalcone; CH, Chioggia; GO, Goro; CE, Cesenatico; SB, San Benedetto; CA, Capoiale.

Specimens of *C. gallina* were collected from six sites in the Western Adriatic Sea from 45°42′N to 41°55′N, spanning ~400 km of latitudinal gradient, ~2°C of average sea surface temperature, ~9 PSU of average sea surface salinity and ~5 mg/m^3^ of average chlorophyll concentration (Figures 1 and 2). Because of the shallow sampling conditions (not beyond 5 m of water depth) and the well mixed water column we assume homogeneous environmental parameters between surface and bottom.

Clams were sampled using hydraulic dredges at 3–5 m depth on sandy or mud bottoms along with the Eastern Italian coasts in six sites from North to South (Figure 1), Monfalcone (MO), Chioggia (CH), Goro (GO), Cesenatico (CE), San Benedetto (SB) and Capoiale (CA). Seawater samples were collected in the six sites in summer (August) and winter (February) in duplicate. In August and February, seawater was the warmest and coldest of the year, respectively, with no sharp shifts in salinity, as occurred during autumn and spring in response to increases rainfall and corresponding river discharge. Although two seawater samples did not allow the capture of seasonal variability over months and years, they can indicate annual seawater isotopes extremes. Seawater samples were stored in plastic jars of 100 ml without additional treatments for δ^18^O and in glass bottles with 1 ml of saturated mercuric chloride (HgCl_2_) for δ^13^C_DIC_, in order to stop all biological activity.

### 2.2 Oxygen and carbon isotopic compositions

For shell δ^18^O and δ^13^C analysis, 7–8 shells from each site were selected and treated with a solution of H_2_O_2_ (10% buffered with ammonium hydroxide) to clean the surface from exogenous sources of oxygen and carbon. Shell powders were manually collected by means of a dental drill (0.5 mm diameter) on the shell surface. The isotope ratios of the shells were determined by using “average shell powder,” which is the combined powders drilled in several points along with the shell growth axis (Figure S1). Seasonal δ^18^O and δ^13^C profiles were carried out on one shell from each of the five sites, by drilling “spot” samples in sequence from the umbo (oldest zone of the shell) to the ventral edge (youngest zone) along with the shell growth axis with ~1.4 mm mean spatial resolution. The necessity of roasting to pyrolize the organic matter before isotopes analyses (350°C for 45 min in vacuo; Keller et al., 2002) was tested by analyzing 12 random powder samples taken from all sites. Shell CaCO_3_ samples of 180–250 μg of powder were flushed with helium gas and reacted with 100% orthophosphoric acid (H_3_PO_4_) and left to equilibrate at 25°C for 24 h. The evolved CO_2_ gas was analyzed using a Finnigan GasBench II connected in line to a Finnigan MAT‐252 isotope ratio mass spectrometer at the Department of Earth and Planetary Sciences, Weizmann Institute of Science. The shell δ^18^O and δ^13^C data are reported against VPDB‐standard.

The analysis of seawater oxygen isotope (δ^18^O_sw_) was performed by mixing 0.5 ml of seawater with 0.5% CO_2_ in helium at 25°C for 24 h. The values are reported in per‐mill relative to the Vienna Standard Mean Ocean Water (VSMOW; ±0.05‰ long‐term precision of laboratory). For carbon isotopes analysis of seawater (δ^13^C_DIC_), 1 ml of seawater was injected into gas vials pre‐flushed with helium and left to react with 0.15 ml H_3_PO_4_ at 25°C for 24 h. The results are reported relative to the international Vienna‐PeeDee Belemnite standard (VPDB; ±0.08‰ long‐term precision of NaHCO_3_ laboratory standard).

Sea surface temperature in each site were reconstructed from shell isotopic composition along with the growth axis and δ^18^O_sw_ to compare with SST data from satellite, by using the equation from Grossman and Ku (1986) [*T* = 20.6 – 4.34 (δ^18^O_arag_ – [δ^18^O_sw_ ‐ 0.27])] (Bemis et al., 1998). In particular, SST were estimated from seasonal δ^18^O_shell_ profile corrected with measured δ^18^O_sw_ (winter, summer and average values for each sites) and with calculated δ^18^O_sw_ reconstructed from salinity data (winter, summer and average sea surface salinity for each sites), using the equation derived from Purroy et al., 2018 for the coastal areas in the eastern Adriatic Sea [δ^18^O_sw_ = 0.23 × salinity ‐ 7.54]. The estimated SSTs were temporally aligned with satellite data, starting from the shell ventral margin corresponding to time of sampling, and backwards sinusoidal sequence of δ^18^O_shell_ defined the seasonal values to consider in the equations (winter for higher peaks, summer for lower peaks and average values for intermediate points).

### 2.3 Diffractometric measurements

X‐ray powder diffraction analyses were carried out in one specimen for each site to determine shell mineral phases. Diffraction patterns were obtained by means of a D2 Phaser diffractometer with Lynxeye detector, using Cu‐Kα radiation generated at 30 kV and 10 mA at the department of Earth and Planetary Sciences in Weizmann Institute of Science. XRD patterns were analyzed using the Diffract.Eva software.

### 2.4 Shell growth parameters

Clam shell length (L, maximum length on the anterior–posterior axis), was calculated with ImageJ software after capturing shell shape with a scanner. Annual growth rates were calculated with the length/age ratio, by using the age obtained from δ^18^O profile along with the shell growth axis.

### 2.5 Environmental parameters

For each site, sea surface temperature (SST; °C) and sea surface salinity (SSS; PSU) data were extrapolated from the database of the Euro‐Mediterranean Center on Climate Change. The annual average of SST and SSS were obtained from daily values from July 2011 to July 2015, while chlorophyll concentration (CHL; mg/m^3^) was calculated from monthly values from the *GlobColour data* by ACRI‐ST, France. The selected range of 4 years for the environmental parameters ensured to enclose the full lifespan of *C. gallina*, reported to be of 2‐3 years in the Adriatic Sea (Mancuso et al., 2019).

### 2.6 Statistical analyses

Significant differences of SST, SSS, CHL, δ^18^O_sw_, δ^13^C_DIC_ and shell δ^18^O and δ^13^C among sites were tested with the one‐way analysis of variance (ANOVA). The non‐parametric Kruskal‐Wallis rank test was used when assumptions for parametric statistics were not fulfilled. The correlations between δ^18^O_sw_ and δ^13^C_DIC_ with latitude were calculated with Spearman's rank correlation coefficient (*r*_*S*_). A General Additive Model (GAM; package mgcv) was used to analyze the influence of different environmental factors on shell δ^18^O and δ^13^C data. GAM are non‐parametric regression techniques that are not restricted by linear relationships, thus they provide a flexible method for analysis when the relationship between variables is complex. GAM was selected based on the gain in deviance explained (%) and on the reduction in Akaike's information criterion (AIC) and generalized cross validation score (GCV) compared to linear model (Table S1). All data analyses were computed using RStudio Software (RStudio Team, 2020).

## 3 RESULTS

### 3.1 Environmental parameters, δ^18^O_sw_ and δ^13^C_DIC_

SST, SSS and CHL from satellite were significantly different among sites in the Adriatic Sea (Kruskal‐Wallis test, *p* < 0.001; Figures 1 and 2). SST and SSS correlated negatively with latitude, while CHL showed the opposite trend.

**FIGURE 3:**
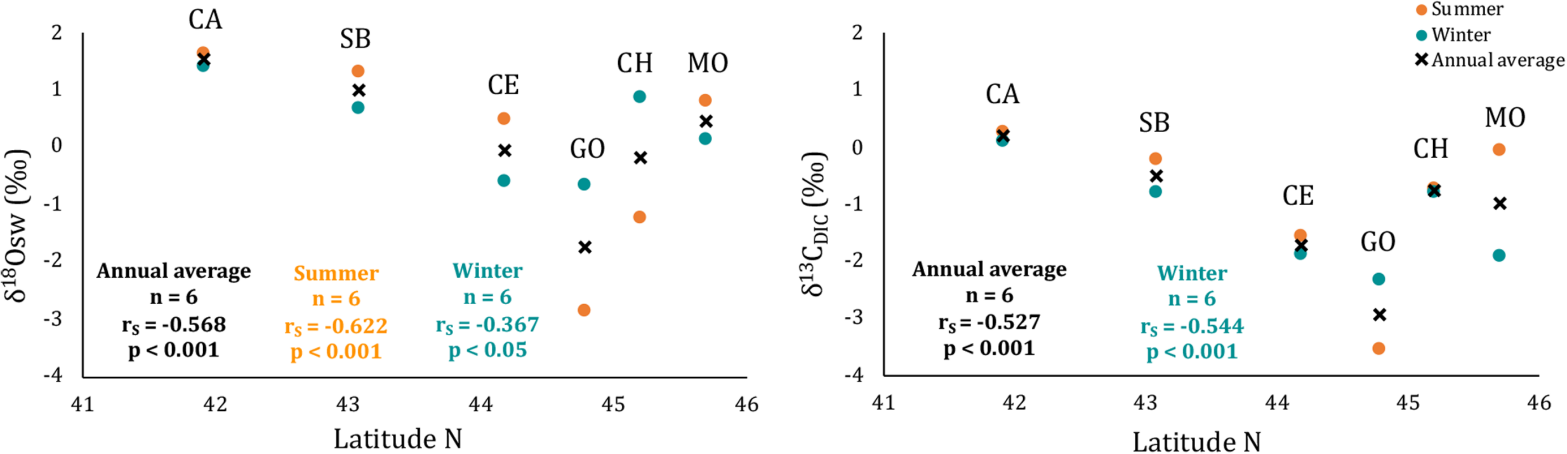
The relation between summer, winter and annual mean δ^18^O_sw_ and δ^13^C_DIC_ with latitude in six sites along with the Western coast of the Adriatic Sea (~400 km transect). Orange dots are summer values, green dots are the winter ones and black crosses are the annual isotope average between summer and winter. No statistics included for summer δ^13^C_DIC_ because of lack of statistically significant. MO, Monfalcone; CH, Chioggia; GO, Goro; CE, Cesenatico; SB, San Benedetto; CA, Capoiale.

Summer, winter and annual mean δ^18^O_sw_ and δ^13^C_DIC_ were significantly different among sites (Kruskal‐Wallis test, *p* < 0.001, Table 1) and correlated negatively with latitude, except for δ^13^C_DIC_ in summer (Figure 3). δ^18^O_sw_ showed positive values while in Chioggia and Goro the δ^18^O_sw_ shifted sharply towards negative values in summer (−1.24‰ and −2.84‰, respectively), even lower than the values in winter (Table 1; Figure 3). In winter, negative δ^18^O_sw_ values were still found in Goro (−0.65‰), together with Cesenatico (−0.60‰) and the resulting annual mean δ^18^O_sw_ showed considerably lower values in Goro (−1.75‰) and marginally lower in Chioggia and Cesenatico (−0.19‰ and −0.06‰, respectively; Table 1; Figure 3). Goro stood out for its deeply low values of δ^13^C_DIC_ in both seasons (−3.54‰ in summer and −2.34‰ in winter), while Capoiale was the only site with positive value of δ^13^C_DIC_, both in summer and winter season (0.28‰ and 0.11‰, respectively; Table 1; Figure 3).

**TABLE 1:** Seawater Isotope data

Site	Latitude (°)	Summer δ^18^O_sw_	Winter δ^18^O_sw_	Annual mean δ^18^O_sw_	Summer δ^13^C_DIC_	Winter δ^13^C_DIC_	Annual mean δ^13^C_DIC_
MO	45.70	0.80	0.12	0.46	−0.05	−1.91	−0.98
CH	45.20	−1.24	0.87	−0.19	−0.73	−0.79	−0.76
GO	44.78	−2.84	−0.65	−1.75	−3.54	−2.34	−2.94
CE	44.18	0.47	−0.60	−0.06	−1.56	−1.88	−1.72
SB	43.08	1.32	0.68	1.00	−0.21	−0.79	−0.50
CA	41.92	1.63	1.41	1.52	0.28	0.11	0.20
K‐W		***	***	***	***	***	***

*Note*: δ^18^O_sw_ (summer, winter and annual average seawater) and δ^13^C_DIC_ (summer, winter and annual average seawater). Values for each site in decreasing order of latitude: MO (Monfalcone), CH (Chioggia), GO (Goro), CE (Cesenatico), SB (San Benedetto), CA (Capoiale). K‐W, Kruskal‐Wallis test; ***p < 0.001.

**FIGURE 4:**
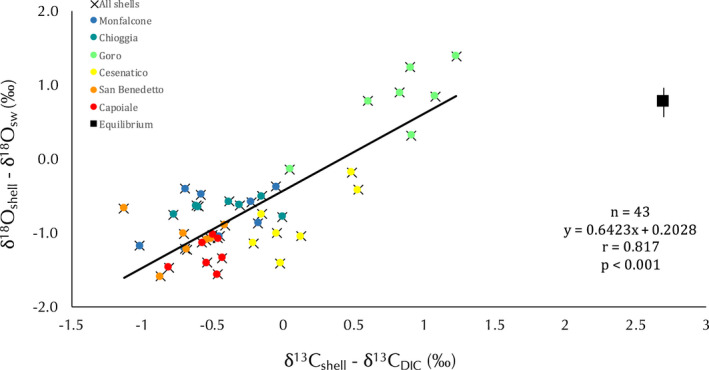
Isotopic comparison among the six sites along with the Adriatic coasts of Italy. The black square depicts the mean estimated aragonite equilibrium value among sites, and the vertical line shows the range of equilibrium values related to local maximum and minimum temperature along with the gradient.

Using the annual mean temperature from all sites along with the latitudinal gradient (17.25°C), the estimated isotopic value for biological aragonite deposited in equilibrium with ambient seawater {calculated from Grossman and Ku (1986) for oxygen [*T* = 20.6 – 4.34 (δ^18^O_arag_ – [δ^18^O_sw_ ‐ 0.27])] and Romanek et al., 1992 for carbon (δ^13^C_arag_ = δ^13^C_DIC_ + 2.7)}, resulted in 0.77‰ ± 0.25‰ for δ^18^O and 2.70‰ for δ^13^C (Figure 4). δ^18^O_sw_ and δ^13^C_DIC_ were not included in the equation as the graph axis are, δ^18^O_shell_ ‐ δ^18^O_sw_ and δ^13^C_shell_ ‐ δ^13^C_DIC_, respectively.

### 3.2 Shell δ^18^O and δ^13^C

**FIGURE 5:**
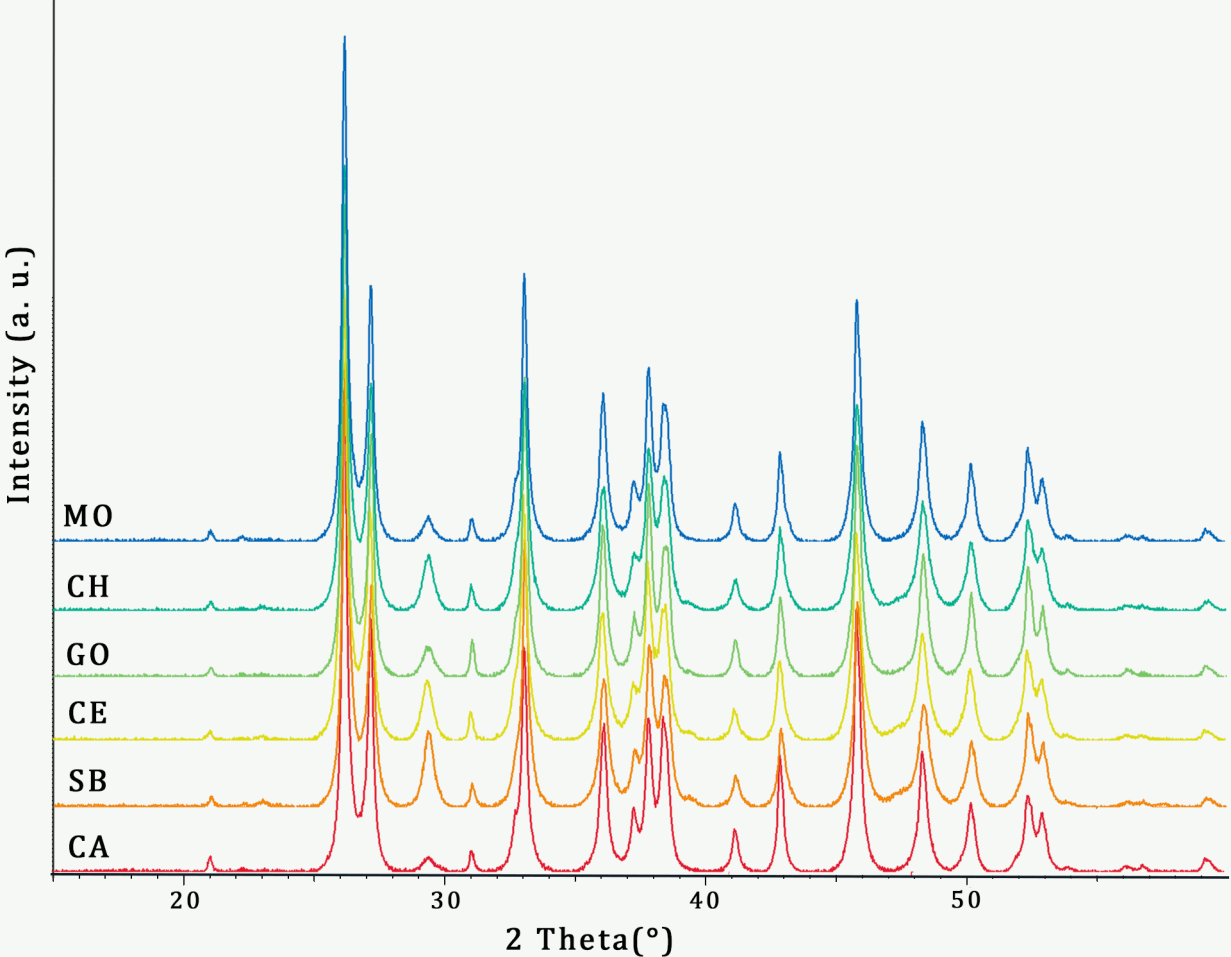
X‐ray powder diffraction (XRD) patterns from ground shells of *C. gallina*. A diffraction pattern is shown for each site. All the peaks were assigned to aragonite.

CaCO_3_ of the analyzed shells of *C. gallina* consisted of aragonite as indicated by XRD patterns obtained for six specimens (one specimen per site; Figure 5). The roasting test showed no differences in the δ^18^O_shell_ and δ^13^C_shell_ between roasted and not roasted powders, with a homogeneous distribution of values obtained from the two procedures (*p* > 0.05; Figure S2). Hence, additional roasting treatment was avoided.

**FIGURE 6:**
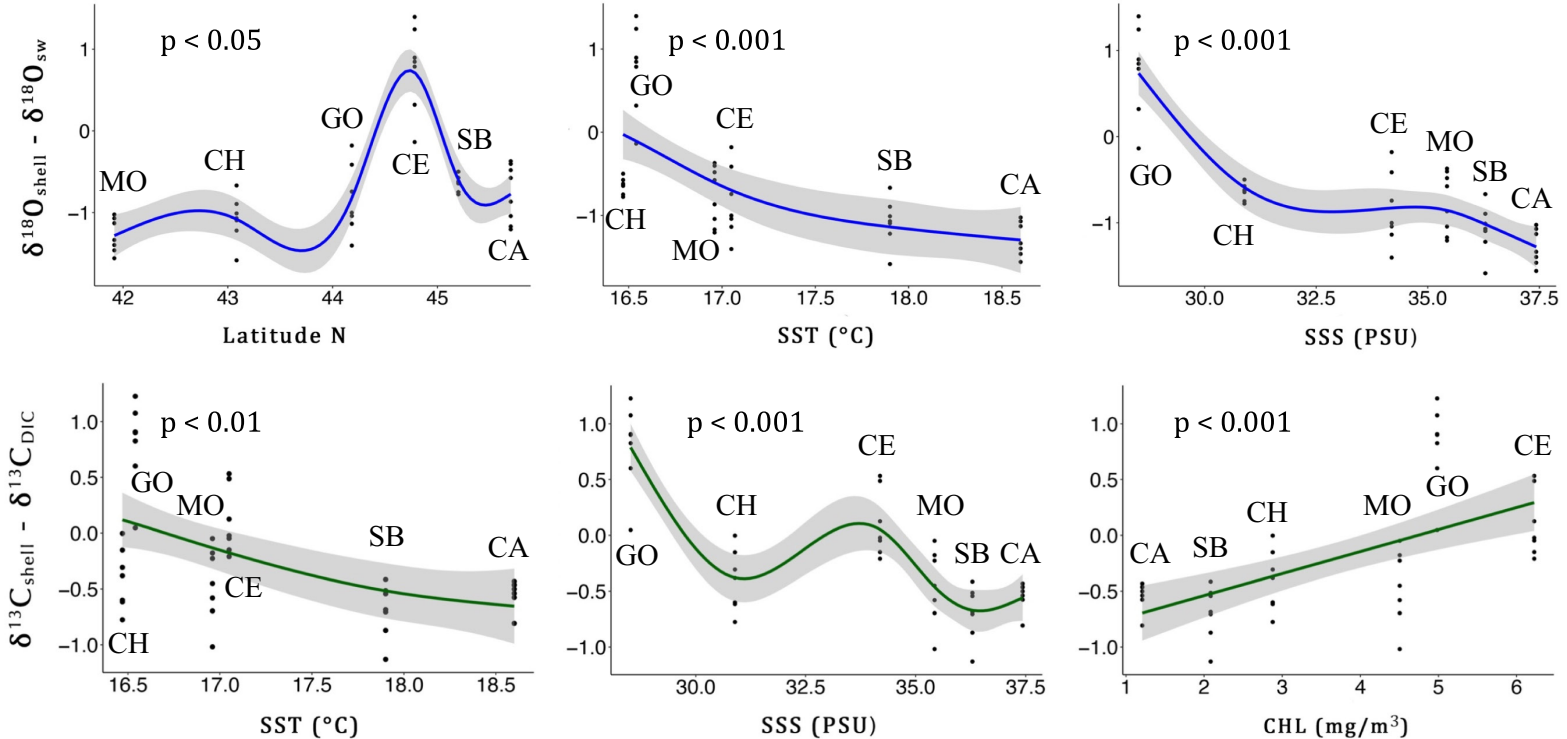
Estimated smooth curve from GAM models. Scatterplots of δ^18^O_shell_ ‐ δ^18^O_sw_ and δ^13^C_shell_ ‐ δ^13^C_DIC_ with fitted smooth terms s(Lat), s(SST), s(SSS), s(CHL) (solid line)*. Number of degrees of freedom = 6* and 95% confidence intervals (grey shade). See Table S2 for statistics.

δ^18^O_shell_ and δ^13^C_shell_ were different among sites (Kruskal‐Wallis test, *p* < 0.001; Table 2). In order to consider the isotopic composition of the local seawater, shell isotope data were corrected with annual mean δ^18^O_sw_ and δ^13^C_DIC_ of each site and the corrected data (indicated as δ^18^O_shell_ ‐ δ^18^O_sw_ and δ^13^C_shell_ ‐ δ^13^C_DIC_) were still different among sites (Kruskal‐Wallis test, *p* < 0.001; Table 2). A strong positive correlation between δ^18^O_shell_ ‐ δ^18^O_sw_ and δ^13^C_shell_ ‐ δ^13^C_DIC_ was observed considering the entire isotope dataset (*p* < 0.001; Figure 4). The measured isotopic values were far from the mean annual estimated aragonite equilibrium value (0.77‰; Figure 4). Only Goro, clustered away from the other sites for both δ^18^O_shell_ ‐ δ^18^O_sw_ and δ^13^C_shell_ ‐ δ^13^C_DIC_, showing δ^18^O_shell_ ‐ δ^18^O_sw_ values close to the aragonite equilibrium value (Figure 4). Moreover, only Goro showed positive δ^18^O_shell_ ‐ δ^18^O_sw_ values, while San Benedetto and Capoiale, the warmer sites, showed lower values (Table 2; Figure 4). δ^18^O_shell_ ‐ δ^18^O_sw_ showed a slight positive correlation with latitude (*p* < 0.05; Table S2; Figure 6), due to higher values reported at Goro (0.76‰), while the two Northern sites, Monfalcone and Chioggia, had lower values (Table 2; Figure 6). δ^18^O_shell_ ‐ δ^18^O_sw_ was also correlated with SST and SSS (*p* < 0.001; Table S2; Figure 6) and with chlorophyll (*p* < 0.05; Table S2; Figure 6).

**TABLE 2:** Shell Isotope data

Site	*n*	δ^18^O_shell_ (SE)	δ^18^O_shell_ ‐ δ^18^O_sw_	δ^13^C_shell_ (SE)	δ^13^C_shell_ ‐ δ^13^C_DIC_
MO	8	−0.31 (0.12)	−0.76	−1.47 (0.11)	−0.49
CH	7	−0.83 (0.04)	−0.64	−1.16 (0.10)	−0.40
GO	7	−0.98 (0.20)	0.76	−2.14 (0.15)	0.80
CE	7	−0.91 (0.16)	−0.85	−1.62 (0.11)	0.10
SB	7	−0.08 (0.11)	−1.08	−1.20 (0.09)	−0.70
CA	7	0.24 (0.08)	−1.28	−0.34 (0.05)	−0.54
K‐W		***	***	***	***

*Note*: SE, standard error of the shell isotope values for each site. KW, Kruskal‐Wallis test; ***p < 0.001. Values for each site in decreasing order of latitude: MO (Monfalcone), CH (Chioggia), GO (Goro), CE (Cesenatico), SB (San Benedetto), CA (Capoiale).

δ^13^C_shell_ ‐ δ^13^C_DIC_ showed no correlation with latitude (Table S2), despite the similar pattern of δ^18^O_shell_ ‐ δ^18^O_sw_, with Goro that presented the highest values (Tables 2 and S2). SST, SSS and CHL showed significant correlations with shell δ^13^C_shell_ ‐ δ^13^C_DIC_ among the six sites (*p* < 0.01 for SST and *p* < 0.001 for SSS and CHL; Table S2; Figure 6).

**FIGURE 7:**
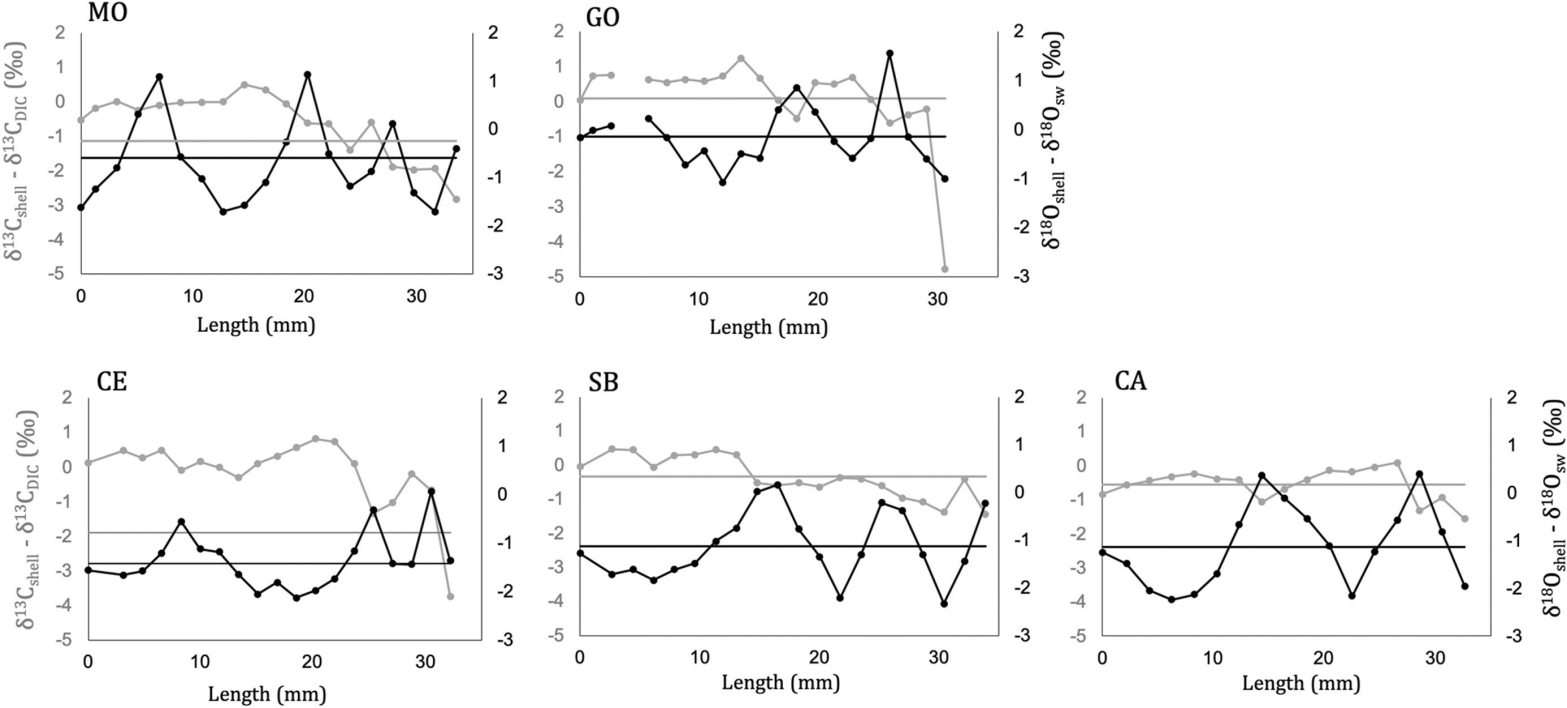
δ^18^O_shell_ ‐ δ^18^O_sw_ and δ^13^C_shell_ ‐ δ^13^C_DIC_ profiles along with the shell growth axis. Grey lines represent δ^13^C, black lines are δ^18^O and points on the lines are the drilled spots. The lower values indicate summer, while the higher values indicate winter. The average values of the drilled spots at each site are reported as horizontal line. No seasonal data for Chioggia.

Seasonal analysis obtained from the drilled spots along with a single shell growth axis revealed that the drilling method, with a mean spatial resolution of ~1.4 mm, resulted in a sinusoidal sequence of lighter and heavier δ^18^O_shell_ with the detection of distinctive seasonal peaks in the δ^18^O_shell_ ‐ δ^18^O_sw_ (Figure 7). The age of five specimens could be defined by counting the sequence of summers (lighter values) and winters (heavier values), and the reduction of the growth rates could be detected with increasing age by the reduction of width of sinusoidal sequences (Figure 7). The shells from Monfalcone and Goro were probably born at the end of summer, while the shells from the other sites were born early in spring or at the beginning of summer, according with the first δ^18^O_shell_ values in each curve (Figure 7).

**FIGURE 8:**
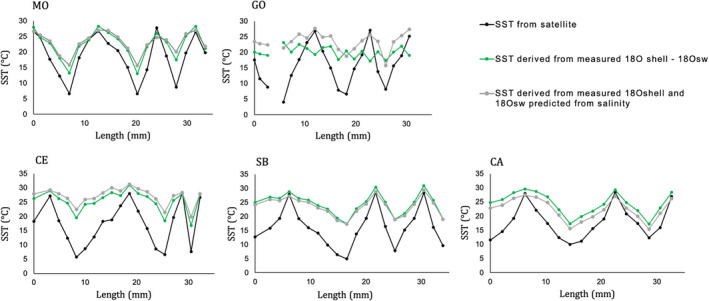
Comparison between SST from satellite and derived SST from oxygen isotopes of *C. gallina*. SST derived from δ^18^O_shell_ along with shell growth axis and measured δ^18^O_sw_ (green line) and predicted δ^18^O_sw_ (grey line), using the equation from Grossman and Ku (1986) [*T* = 20.6 – 4.34 (δ^18^O_arag_ ‐ [δ^18^O_sw_ ‐ 0.27])]. Winter, summer or average δ^18^O_sw_ were considered depending on seasonal profile observed in the sinusoidal sequence of oxygen isotopes during shell growth. Predicted δ^18^O_sw_ was calculated from salinity data (grey line) using the equation derived from Purroy et al., (2018) [δ^18^O_sw_ = 0.23 x salinity ‐ 7.54]. Winter, summer or average salinity SST obtained from satellite data (black line).

Sea surface temperature derived from measured δ^18^O_shell_ and δ^18^O_sw_ reflected the SST from satellite during warm seasons in all sites except in Goro, where only SST calculated with δ^18^O_sw_ predicted from summer salinity data aligned with summer SST from satellite (Figure 8). In the cold season SST derived from both measured δ^18^O_sw_ and predicted δ^18^O_sw_ from salinity seemed to overestimate the temperature in all sites (Figure 8). In Goro, reconstructed SST from δ^18^O_sw_ results are inconsistent with winter seasonal peaks (Figure 8). In general, the two methods used for obtaining SST from measured δ^18^O_sw_ and from predicted δ^18^O_sw_ from salinity agree, showing similar SST in five sites, except in Goro (Figure 8). In Goro, SST derived from measured δ^18^O_sw_ showed no seasonal profile, suggesting an apparent uncertainty in the measured δ^18^O_sw_ values in this site (Figure 8).

**FIGURE 9:**
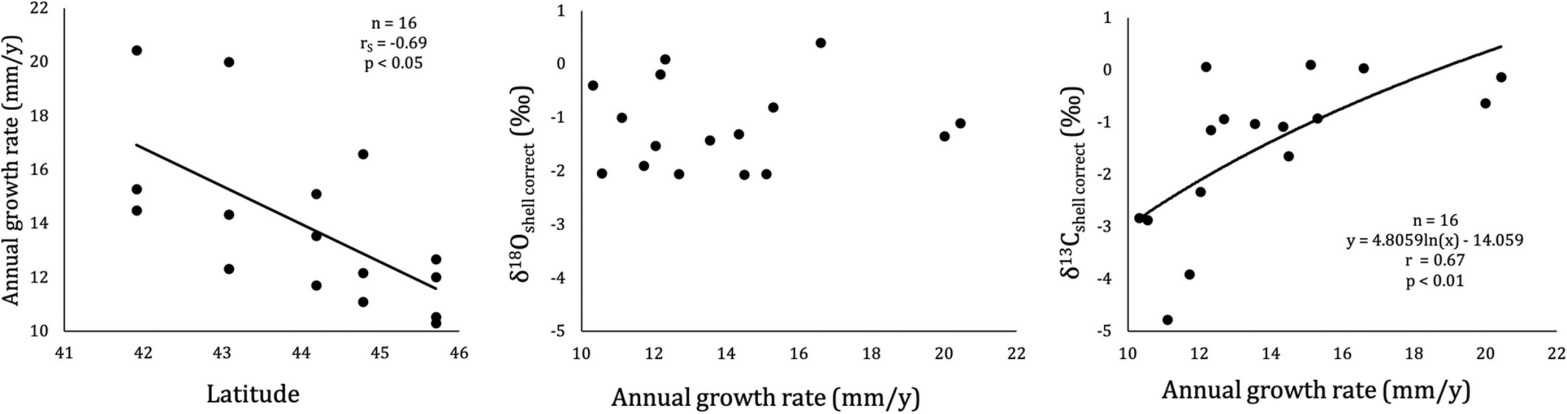
Relationship between annual growth rates with latitude, δ^18^O_shell_ ‐ δ^18^O_sw_ and δ^13^C_shell_ ‐ δ^13^C_DIC_. Growth rates are calculated from the length‐age key at each year, by means of δ^18^O profile along with the shell growth axis.

δ^13^C_shell_ ‐ δ^13^C_DIC_ of *C. gallina* differed within the shells and among sites, showing a decreasing trend from the umbo to the ventral edge in all shells and higher variability for δ^13^C_shell_ ‐ δ^13^C_DIC_ in the shells from the Northern sites (Figure 7). The large variability in δ^13^C_shell_ ‐ δ^13^C_DIC_ was observed especially over 30 mm in the shells of Goro and Cesenatico, where considerable peaks of reduced δ^13^C_shell_ ‐ δ^13^C_DIC_ were depicted (−4.17‰ and −3.91‰, respectively; Figure 7). Positive correlation was found between shell carbon isotope values and annual growth rate (Figure 9).

## 4 DISCUSSION

Shell δ^18^O and δ^13^C of the clam *C. gallina* and seawater oxygen and carbon isotope signatures were investigated for the first time under varying temperature, salinity, and chlorophyll concentration conditions along with a wide latitudinal gradient in the Adriatic Sea. The Po river inflows in the Northern Adriatic Sea and heavily modifies salinity and chlorophyll concentrations from North to South (Catalano et al., 2014; Gilmartin et al., 1990). Po river is responsible for 50% of the total nutrient input (Pettine et al., 1998), that gives rise to phytoplankton blooms in spring, making the Northern Adriatic Sea the area with the highest average primary production in the Adriatic basin (588 g C m^2^ y^1^; Gilmartin et al., 1990). In contrast, in the Middle and Southern Adriatic Sea primary production is significantly lower (137 and 97 g C m^2^ y^1^, respectively), resulting in a relevant eutrophic/oligotrophic gradient along with the Eastern coasts of Italy from North to South (Giordani et al., 2002).

The biology of the clam *C. gallina* in the Adriatic Sea was already studied in terms of growth rate (Bargione et al., 2020; Keller et al., 2002; Mancuso et al., 2019), physiology (Matozzo et al., 2007; Monari, Matozzo, et al., 2007; Monari, Serrazanetti, et al., 2007), and shell properties (Cheli et al., 2021; Gizzi et al., 2016; Mancuso et al., 2019). A previous study conducted in the Bay of Trieste in the North of the Adriatic Sea investigated the shell isotopic composition of *C. gallina* in relation to its growth rates and settlement time (Keller et al., 2002). However, this confined area is not representative of the wide shifts in *C. gallina* habitat from North to South of the Adriatic basin that influence shell growth (Mancuso et al., 2019).

### 4.1 δ^18^O_sw_ and δ^13^C_DIC_

In this study, both δ^18^O_sw_ and δ^13^C_DIC_ varied along with the ~400 km transect in the Adriatic Sea. The annual δ^18^O_sw_ varied by 3.27‰ VSMOW along with the latitudinal gradient, in contrast to the remarkably constant δ^18^O_sw_ along with the 850 km North–South transect in the Italian Western coast (0.65‰ ± 0.23 SD; Prada et al., 2019). The main driver controlling δ^18^O_sw_ in this region is the increase of freshwater inflows and the large salinity changes, from ca. 29 to 37 PSU, from the area around the Po delta towards the South. Freshwater mixing from the Po river (δ^18^O close to −10‰; Bortolami et al., 1973) leads to low salinity conditions (<30 PSU) and extremely low δ^18^O_sw_ values (−1.75‰) in Goro. The Po is dominated by two annual floods due to raised rainfall in autumn and snow‐melt in spring (Flora & Longinelli, 1989; Tesi et al., 2007) and largest minimum, largest average and largest maximum daily river flow are observed to be 275, 1470 and 10,300 m^3^ s^−1^, respectively (Montanari, 2012). The freshwater gain along with the coastline of the Northern basins results in a negative difference between the freshwater losses by evaporation and the gains by runoff and precipitation, making the Adriatic Sea a dilution basin compared to the Mediterranean Sea as a whole (Raicich, 1996). Moreover, the surface circulation of the Adriatic Sea is primarily thermohaline, driven by dense water formation related to the surface heat losses: cool and with low salinity North Adriatic dense Deep Water (NAdDW), that flows southwards and Southern Adriatic dense Deep Water (SAdDW), favored by cyclonic gyre (Russo & Artegiani, 1996; Zavatarelli et al., 1998). Positive δ^18^O_sw_ values were reported in the Southern sites (1.52‰ in Capoiale), likely due to the supply of saltier and isotopically heavier seawater from the Southern Adriatic, such as the Levantine Intermediate Water (LIW) that flows northwards in the Adriatic through the Otranto Strait (Stenni et al., 1995). δ^13^C_DIC_ values also strongly varied along with the latitudinal gradient with a range of 3.14‰, compared to the Eastern and Western Mediterranean δ^13^C_DIC_ values, 0.41‰ and 0.43‰, respectively (Pierre, 1999). δ^13^C_DIC_ increased from North (−0.98‰ in Monfalcone) to South (0.20‰ in Capoiale) with a sharp depleted value in the Po delta area (−2.94‰ in Goro). δ^13^C_DIC_ along with the gradient reflected the contribution of isotopically light carbon from freshwater inflow (δ^13^C_DIC_ close to −10.5‰ ±0.4; Marchina, 2015), from sites around the Po delta.

### 4.2 Shell δ^18^O

Along with the latitudinal gradient, temperature and salinity covary and their decrease towards Northern sites led to an δ^18^O_shell_ ‐ δ^18^O_sw_ increase. Indeed, at thermodynamic equilibrium, δ^18^O_shell_ depends on the precipitation temperature and seawater δ^18^O (Craig, 1965; Epstein et al., 1951; Grossman & Ku, 1986). δ^18^O_shell_ ‐ δ^18^O_sw_ also reflected differences in δ^18^O ranging from 0.76‰ in Goro to −1.28‰ in Capoiale. While the site of Goro was close to the expected oxygen isotopic equilibrium (0.77‰), the other five sites showed a negative offset from equilibrium, with the largest offset in Capoiale (−2.05‰). The δ^18^O_shell_ ‐ δ^18^O_sw_ fluctuation of 2.04‰ between Goro and Capoiale would require temperature variations of about 9°C (Craig, 1965; Grossman & Ku, 1986) if temperatures were the only cause of the δ^18^O_shell_ ‐ δ^18^O_sw_ fluctuation. δ^18^O values are also changeable in relation to salinity of the habitat (Craig, 1965) and high fluctuations of salinity in Goro, the site close to Po delta, were likely responsible for the deviation of temperature derived from measured δ^18^O_shell_ ‐ δ^18^O_sw_, that did not reflect the annual fluctuation of seawater temperature. The two Northern sites, Monfalcone and Chioggia, had lower values of δ^18^O_shell_ ‐ δ^18^O_sw_, suggesting a minor influence of Po freshwater mixing and a contribution of heavier δ^18^O_sw_ from the Eastern Adriatic coast.

Temperatures reconstructed from δ^18^O_shell_ ‐ δ^18^O_sw_ showed that higher SST agreed well with real summer SST, while lower SST was consistently higher than the real winter SST obtained from satellite data. This inconsistency could be attributed to the fact that *C. gallina* precipitates the shell carbonate preferentially during the warm period, while considerably reducing its activity during the cold season (Cespuglio et al., 1999; Keller et al., 2002). The same seasonality of shell growth in the Adriatic Sea was found in the long‐lived bivalve *Glycymeris pilosa* and in the venerid *Callista chione* and *Venus verrucosa*, that showed to be a promising archive for the reconstruction of summer seawater temperatures with the slowest growth during winter (Peharda et al., 2019; Purroy et al., 2018; Uvanović et al., 2021). Shell deposition with warm temperatures was reported also for other bivalves from different parts of the globe, such as *Mercenaria stimpsoni*, *Chione cortezi* and *Phacosoma japonicum*, showing that these shells cannot be used as archives of winter temperatures (Goodwin et al., 2001; Kubota et al., 2017; Tanabe & Oba, 1988). In agreement with these previous results, *C. gallina* might be a warm season temperature proxy, suggesting additional complexity in utilizing *C. gallina* shells in paleoclimate studies. Indeed, when shell growth rate is not constant during the year, growth suspensions hamper bivalves from providing uninterrupted records of environmental conditions (Goodwin et al., 2003).

It has been suggested that kinetic effects, associated with the hydration reaction and carbonate biomineralization, during higher calcifications rates increase the relative amount of ^16^O incorporated in the newly formed shell, resulting in more negative δ^18^O_shell_ signatures than equilibrium values (McConnaughey, 1989a). The shells of Southern sites (with higher growth rates; Mancuso et al., 2019), showed the larger oxygen isotopic offset from expected equilibrium, suggesting that together with temperature and salinity, kinetic effects could also explain this observed departure. As bivalves mostly use the oxygen of the ambient water (HCO_3_^−^) rather than ingested food for shell growth (Epstein & Mayeda, 1953; McConnaughey, 1989a), metabolic isotope effects related to respiration can be excluded.

From δ^18^O_shell_ ‐ δ^18^O_sw_ profiles along with the *C. gallina* growth direction we could derive considerations on growth rates at the investigated sites in the Adriatic Sea. δ^18^O_shell_ ‐ δ^18^O_sw_ profiles indicated that samples of Cesenatico, San Benedetto and Capoiale were born in spring, while the samples of Monfalcone and Goro, with colder water, were born later in summer. A notable reduction in growth rates with increasing length was also observed in the decreasing amplitude of sinusoidal sequence with clam size. By counting age from seasonal δ^18^O_shell_ ‐ δ^18^O_sw_ peaks, *C. gallina* reached a length of about 20 mm after 1 year in the Southern sites (San Benedetto and Capoiale), and about 13 mm after 1 year in the Northern site (Monfalcone), indicating higher linear extension rates towards the South, in agreement with the previous study (Mancuso et al., 2019).

### 4.3 Shell δ^13^C

δ^13^C_shell_ ‐ δ^13^C_DIC_ exhibited a ~ 1.5‰ range with the largest difference between Goro (0.80‰) and San Benedetto sites (−0.69‰). Seawater δ^13^C_DIC_ and metabolic carbon from bivalve respiration and diet significantly affect shell δ^13^C values (Gillikin et al., 2006; Lorrain et al., 2004; McConnaughey, 1989a, 1989b). In this study, δ^13^C_shell_ ‐ δ^13^C_DIC_ was not correlated with latitude, unlike δ^13^C_DIC_, implying the contribution of metabolic carbon in *C. gallina* shells. Molluscs shells in coastal areas incorporate carbon from both the riverine and marine reservoirs, so δ^13^C_shell_ reflects the mixture (Gillikin et al., 2006; Mook & Vogel, 1968). In this study, the δ^13^C_shell_ ‐ δ^13^C_DIC_ of *C. gallina* increased with decreasing salinity and increasing chlorophyll concentration, perhaps as a result of decreasing calcification. *C. gallina* shows reduced calcification rate with increasing chlorophyll concentration along with the same gradient, perhaps as a result of increased sedimentation as a result of river discharges in proximity to the Po delta, which could negatively impact the feeding mechanisms of the clams (Mancuso et al., 2019; McConnaughey & Gillikin, 2008; Pérez et al., 2016).

δ^13^C_shell_ ‐ δ^13^C_DIC_ profiles along with the growth axis showed lower values with increasing length, more pronounced in the Northern sites, with Goro showing the larger ontogenetic variability (up to 4.8‰) between the umbo (the older part of shells) and the ventral margin (the youngest part of shells). In previous studies, the general decreasing trend of δ^13^C through ontogeny was observed to be either caused by the influence of pore water δ^13^C_DIC_ gradients, or effects of metabolic changes (Elliot et al., 2003; Gillikin et al., 2007; Krantz et al., 1987; Lorrain et al., 2004). Indeed, a deeper position in the sediment of older specimens seemed to lead to an increased supply of ^13^C depleted pore water produced by the oxidation of organic matter or to the incorporation of larger amounts of respiratory CO_2_ (Keller et al., 2002; Lorrain et al., 2004; McConnaughey & Gillikin, 2008). Infaunal bivalves may show isotopically depleted values compared to epifaunal species (Keller et al., 2002; Krantz et al., 1987). Another possible explanation could be the variation of the metabolism associated with shell growth rates. Rosenberg & Hughe, 1991 found lower mantle metabolic activity in faster growing shell portions in *Mytilus edulis*. Lorrain et al., 2004 reported a reduction in the δ^13^C_shell_ of *Pecten maximus* as result of increasing utilization of ^13^C depleted respiratory CO_2_ through ontogeny. Similarly, higher metabolic rates needed to support carbon requirements for calcification could explain the decrease of δ^13^C_shell_ ‐ δ^13^C_DIC_ through ontogeny observed in *C. gallina*. The strong link between carbon isotopes and bivalve metabolic activity suggests that carbon isotopes are not reliable indicators of environmental conditions in biogenic carbonates given the high variability of metabolic carbon across seasons, shell growth rates and ontogenetic variations (Geist et al., 2005; Gillikin et al., 2006; Lorrain et al., 2004).

*Chamelea gallina* showed a negative offset of 1.9‰ in Goro and 3.4‰ in San Benedetto from the expected carbon isotopic equilibrium (2.7‰), in agreement with most benthic species that generally have depleted δ^13^C values compared to carbon isotopic equilibrium (Keller et al., 2002; Rau et al., 1982). The decrease δ^18^O_shell_ and δ^13^C_shell_ with respect to the equilibrium was in agreement with the classical models driven by kinetic effects, that lead to isotope depleted carbonates (Adkins et al., 2003), highlighting shell stable isotope vital effects in *C. gallina* and contributing to set limits for paleoenvironmental reconstructions for this species.

## 5 CONCLUSIONS

Bivalve shells can potentially provide information about past estuarine biogeochemical cycles by recording the carbon isotopic signature of dissolved inorganic carbon (δ^13^C_DIC_) in estuarine waters. The Adriatic Sea, and especially its Northern basin, plays an important role in carbon cycling being a site of dense water formation during winter and one of the most productive areas in the Mediterranean, contributing to global biogeochemical cycling of carbon and nutrients (Catalano et al., 2014; Crossland et al., 2005). Here we present, for the first time, measurements of shell δ^18^O and δ^13^C of *C. gallina* combined with seawater δ^18^O and δ^13^C_DIC_ along with the 400 km Western Adriatic coasts. The high variability of seawater parameters was expressed in the stable isotopic signature of *C. gallina* along with the latitudinal gradient. *Chamelea gallina* from Northern sites clearly reflected lower temperature of deposition and the influence of Po river, while shells from Southern sites reflected the salty marine ingressions from the Southern Adriatic. Shells displayed depleted δ^13^C values with decreasing salinity and increasing chlorophyll concentration, likely as a result of decreased calcification rates likely due to increased eutrophication and silt and clay of the bottom driven by the river discharges. Almost all specimens exhibited depleted shell δ^18^O and δ^13^C values compared to the expected isotopic equilibrium. Hence, despite *C. gallina* showing promise as a warm temperature proxy, the large variation in the shell stable isotopic signature points toward noteworthy metabolic and/or kinetic effects in this bivalve, preventing the use of *C. gallina* as a paleoproxy for seawater temperatures.

## AUTHOR CONTRIBUTION

**Arianna Mancuso** involved in conceptualization, methodology, formal analysis, investigation, resources, writing the original draft, and visualization. **Ruth Yam** involved in methodology, formal analysis, investigation, resources, review and editing, and visualization. **Fiorella Prada** involved in review and editing, and visualization. **Marco Stagioni** involved in methodology, resources, review, and editing. **Stefano Goffredo** involved in conceptualization, resources, review and editing, and supervision. **Aldo Shemesh** involved in conceptualization, methodology, resources, review and editing, and supervision.

## FUNDING INFORMATION

This research did not receive any specific grant from funding agencies in the public, commercial, or not‐for‐profit sectors.

## CONFLICT OF INTEREST

The authors declare that they have no competing interests.

## DATA AVAILABILITY STATEMENT

The dataset generated and analyzed during the current study is available from the corresponding author on reasonable request.

## Supporting information


Appendix S1
