# Supplementary material for: Oxygen and carbon isotope variations in Chamelea gallina shells: Environmental influences and vital effects
Source: Geobiology. 2022 Sep 26;21(1):119–32. doi: 10.1111/gbi.12526 (PMC10087952; doi:10.1111/gbi.12526)
Supplement: Supplementary file 1 — Appendix S1 [file GBI-21-119-s001.docx]

**Supporting information**

**Oxygen and carbon isotopes variations in the bivalve shell of *Chamelea gallina:* environmental influences and vital effects**

**Table S1. Comparison between fitting data with linear model and GAM.**

Three measures of performance were considered: adjusted R-squared, Akaike’s information criterion (AIC) and generalized cross validation score (GCV). AIC and GCV are similar comparative measures to choose among different models, with lower values being better. Examing these indices, it’s safe to conclude that the GAM performs considerably better compared to LM.

|  |  | **δ^18^O_shell_ - δ^18^O_sw_** | | | **δ^13^C_shell_ - δ^13^C_DIC_** | | |
| --- | --- | --- | --- | --- | --- | --- | --- |
|  |  | AIC | GCV | r2 | AIC | GCV | r2 |
| **Latitude** | LM | 91.602 | 0.471 | 0.174 | 77.126 | 0.337 | 0.047 |
|  | GAM | 38.058 | 0.138 | 0.781 | 22.392 | 0.096 | 0.754 |
| **Temperature** | LM | 80.735 | 0.366 | 0.359 | 68.560 | 0.276 | 0.219 |
|  | GAM | 38.044 | 0.138 | 0.781 | 68.155 | 0.274 | 0.236 |
| **Salinity** | LM | 56.272 | 0.207 | 0.637 | 52.151 | 0.188 | 0.467 |
|  | GAM | 36.354 | 0.132 | 0.783 | 21.613 | 0.094 | 0.756 |
| **Chlorophyll** | LM | 91.132 | 0.466 | 0.183 | 61.689 | 0.235 | 0.334 |
|  | LM | 38.052 | 0.138 | 0.781 | 22.394 | 0.096 | 0.754 |

**Table S2. Generalised Additive Model (GAM) results.** GAM describing the correlation of response of δ^18^O and δ^13^C with latitude, s(Lat); sea surface temperature s(SST); salinity s(SSS); chlorophyll concentration s(CHL). Edf, degrees of freedom estimated for the model; F, statistical test to evaluate the significance of smoothed terms. *p<0.05; **p<0.01; ***p<0.001. GCV, generalized cross validation score. Deviance explained represents the reduction in the deviance achieved by the models, expressed as a percentage of the deviance of the null model (null deviance).

| **GAM δ^18^O** | **Edf** | **F** | **p-value** | **GAM δ^13^C** | **Edf** | **F** | **p-value** |
| --- | --- | --- | --- | --- | --- | --- | --- |
| s(Lat) | 1.4 | 4.9 | 0.011*... | s(Lat) | 1.0 | 2.3 | 0.139.... |
| s(SST) | 1.3 | 13.6 | 0.001*** | s(SST) | 1.0 | 11.3 | 0.002**. |
| s(SSS) | 1.0 | 43.2 | 0.000*** | s(SSS) | 1.0 | 36.7 | 0.000*** |
| s(CHL) | 1.0 | 6.2 | 0.015*.. | s(CHL) | 1.0 | 48.5 | 0.000*** |
|  |  |  |  |  |  |  |  |
| Adjusted R^2^ | 0.781 | Deviance explained | 80.60% | Adjusted R^2^ | 0.761 | Deviance explained | 78.30% |
| GCV score | 0.138 | n | 43 | GCV score | 0.091 | n | 43 |

**
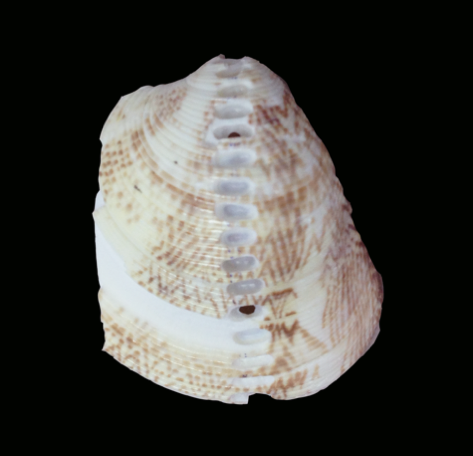
**

**Fig. S1.** **Samples of *Chamelea gallina* with drilling “spot”, from the umbo to the ventral edge along the shell growth axis.**

**
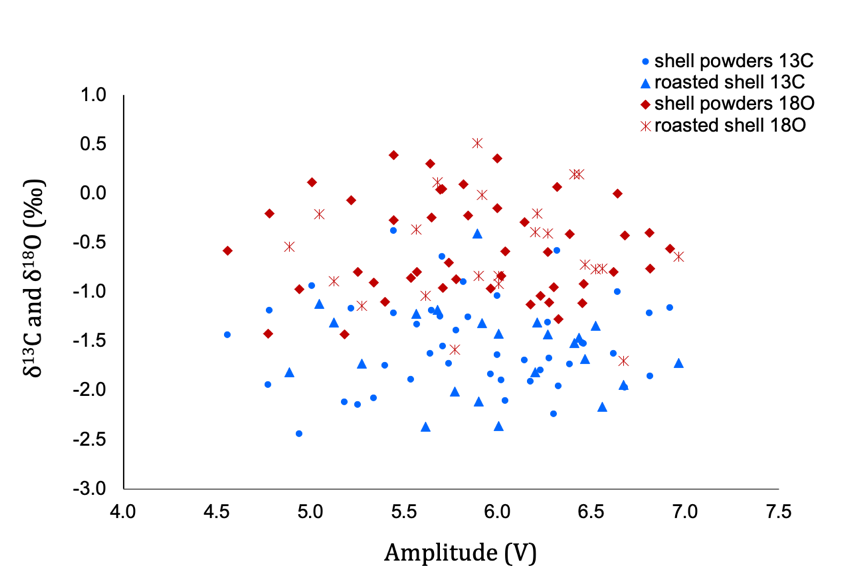
**

**Fig. S2. Shell δ^13^C and δ^18^O values vs Amplitude (V**). n=12 roasted and 12 not roasted samples.
